# Regulation of BK channels by auxiliary γ subunits

**DOI:** 10.3389/fphys.2014.00401

**Published:** 2014-10-15

**Authors:** Jiyuan Zhang, Jiusheng Yan

**Affiliations:** Department of Anesthesiology and Perioperative Medicine, University of Texas MD Anderson Cancer CenterHouston, TX, USA

**Keywords:** BK channels, KCNMA1, Slo1, K_Ca1.1_, auxiliary subunit, accessory protein, regulation, modulation

## Abstract

The large-conductance, calcium- and voltage-activated potassium (BK) channel has the largest single-channel conductance among potassium channels and can be activated by both membrane depolarization and increases in intracellular calcium concentration. BK channels consist of pore-forming, voltage- and calcium-sensing α subunits, either alone or in association with regulatory subunits. BK channels are widely expressed in various tissues and cells including both excitable and non-excitable cells and display diverse biophysical and pharmacological characteristics. This diversity can be explained in part by posttranslational modifications and alternative splicing of the α subunit, which is encoded by a single gene, *KCNMA1*, as well as by tissue-specific β subunit modulation. Recently, a leucine-rich repeat-containing membrane protein, LRRC26, was found to interact with BK channels and cause an unprecedented large negative shift (~-140 mV) in the voltage dependence of the BK channel activation. LRRC26 allows BK channels to open even at near-physiological calcium concentration and membrane voltage in non-excitable cells. Three LRRC26-related proteins, LRRC52, LRRC55, and LRRC38, were subsequently identified as BK channel modulators. These LRRC proteins are structurally and functionally distinct from the BK channel β subunits and were designated as γ subunits. The discovery of the γ subunits adds a new dimension to BK channel regulation and improves our understanding of the physiological functions of BK channels in various tissues and cell types. Unlike BK channel β subunits, which have been intensively investigated both mechanistically and physiologically, our understanding of the γ subunits is very limited at this stage. This article reviews the structure, modulatory mechanisms, physiological relevance, and potential therapeutic implications of γ subunits as they are currently understood.

## Discovery of γ subunits

**Figure 1:**
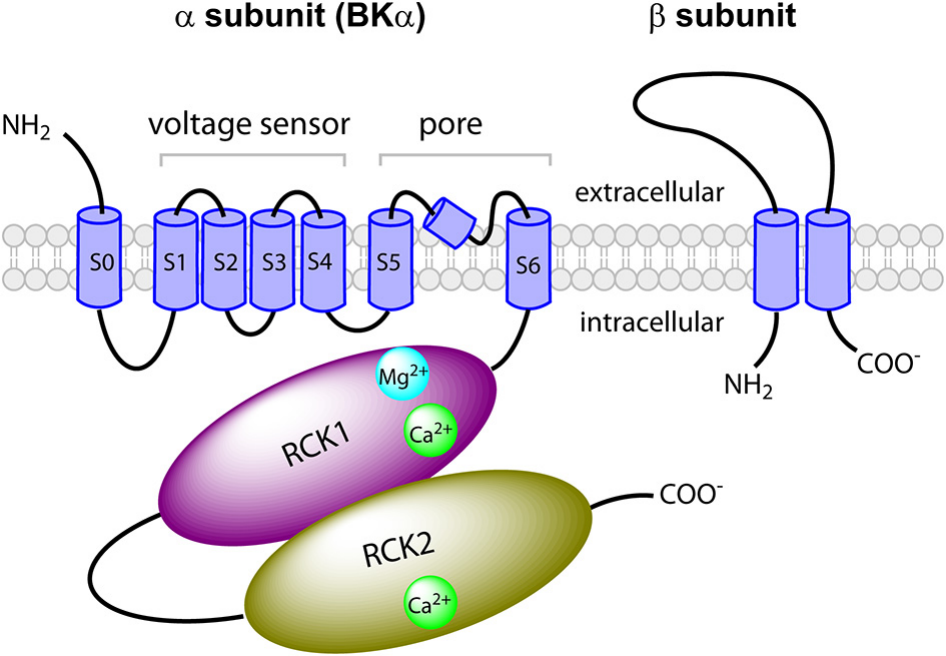
**Schematic structure and membrane topology of BK channel α and β subunits**.

Among the numerous K^+^ channels, the large-conductance Ca^2+^- and voltage-activated K^+^ channel (termed BK, Slo1, K_*Ca*1.1_, or KCNMA1; hereafter BK) is considered unique, characterized by its large single-channel conductance and dual activation by membrane depolarization and elevation in intracellular free calcium ([Ca^2+^]_i_) (Marty, 1981; Latorre and Miller, 1983; Golowasch et al., 1986; Latorre et al., 1989). The structure, function, and regulatory mechanisms of BK channels have been investigated over the past 3 decades. BK channels are considered channel complexes composed of either homotetramers of the pore-forming and calcium- and voltage-sensing α subunit (BKα) alone or BKα together with tissue-specific auxiliary subunits. BKα is structurally distinct from most other K^+^ channels because it possesses an extra *N*-terminal transmembrane segment (S0) (Wallner et al., 1996; Meera et al., 1997) and a large Ca^2+^-sensing cytosolic *C*-terminus composed of two RCK (regulating conductance of K^+^) domains (Wu et al., 2010; Yuan et al., 2012) (Figure 1). BKα is also distinct from most other voltage-gated K^+^ (Kv) channels in the pore-forming transmembrane S1–S6 domains by a very low amino acid sequence similarity with them.

BK channels occur in many different tissues and cells and display diverse biophysical or pharmacological characteristics. This diversity can be explained in part by posttranslational modifications and alternative splicing of the large BKα subunit (~130 kDa), which is encoded by a single gene, *KCNMA1*, as well as by tissue-specific β subunit modulation. Four different β subunits (β1–β4) have been cloned and identified in mammals. The first β subunit was identified as a binding partner of BKα in the BK channel complexes purified from bovine tracheal smooth muscle by extensive conventional chromatography together with sucrose gradient centrifugation or by immunoprecipitation (Knaus et al., 1994a,b,c). In these early experiments, charybdotoxin (ChTx), which is a specific peptide blocker of BK channels, was radiolabeled as a tool for BK channel complex or protein detection and ChTx was found to be attached to a β subunit upon cross-linking. This smooth muscle–specific auxiliary protein was later named the β1 subunit, and the other 3 family members were discovered thereafter (Wallner et al., 1999; Xia et al., 1999; Behrens et al., 2000; Brenner et al., 2000; Meera et al., 2000; Uebele et al., 2000).

Each β subunit has 2 transmembrane segments connected by a large loop on the extracellular side (Figure 1). The 4β subunits display different and complex effects on apparent calcium and voltage sensitivities, macroscopic current kinetics, and pharmacological sensitivities, which involve multiple distinct mechanisms. For example, the β1 and β2 subunits overall induce slowing of the macroscopic kinetics and an increase in apparent calcium and voltage sensitivity (Behrens et al., 2000; Brenner et al., 2000; Savalli et al., 2007; Contreras et al., 2012). The β2 and some splice variants of β3 subunits also cause rapid inactivation through their intracellular *N*-termini (Wallner et al., 1999; Xia et al., 1999, 2000; Uebele et al., 2000). The β3 subunits generate rectifying outward currents regulated by their extracellular loops (Xia et al., 2000; Zeng et al., 2003). The brain-specific β4 subunit, in addition to greatly slowing activation and deactivation kinetics, reduces apparent calcium sensitivity in low [Ca^2+^]_i_ conditions but increases apparent sensitivity in high [Ca^2+^]_i_ conditions (Behrens et al., 2000; Brenner et al., 2000).

The wide distribution of BKα suggests that BK channels have a potentially important function in various physiological processes (Nelson et al., 1995; Hu et al., 2001; Gu et al., 2007). The activation mechanisms and functions of BK channels in excitable neuronal and smooth muscle cells have been intensively studied and are relatively well understood in principle, although more remains to be examined, such as channel heterogeneity caused by different auxiliary subunit composition in different cells. Compared with other voltage-gated or Ca^2+^-activated K^+^ channels, BK channels have much higher thresholds for channel activation by either voltage or [Ca^2+^]_i_ alone; these thresholds are generally outside of physiological ranges. Thus, in excitable cells, activation of BK channels typically requires coincident membrane depolarization and elevation in [Ca^2+^]_i_ levels (Brenner et al., 2000; Salkoff et al., 2006), and BK channels are physically coupled to voltage-gated Ca^2+^ channels in order to sense locally enriched Ca^2+^ (Berkefeld et al., 2006; Fakler and Adelman, 2008). However, non-excitable cells generally have a relatively constant low resting membrane potential and a marginal amount of [Ca^2+^]_i_. BK channel function and mechanism of activation has been largely unexplored in non-excitable cells.

In 2005, an unusal type of K^+^ current was reported in lymph node carcinoma of the prostate (LNCaP) cells, which showed a Kv-like low half-activation voltage (V_1/2_) of ~30 mV in the absence of [Ca^2+^]_i_ but had many characteristics of BK chanenls (Gessner et al., 2005). These BK-like features included large single-channel conductance (220 pS at 140 mM symmetric K^+^), activation by [Ca^2+^]_i_ in the μM range and [Mg^2+^]_i_ in the mM range, and sensitivity to specific activator NS1619 and blockers ChTx, IbTx, paxilline, and penitrem A (Gessner et al., 2005). The voltage dependence of channel activation for this endogenous BK-like channel in LNCaP cells was shifted to the hyperpolarization direction by more than 120 mV compared with human BKα channels expressed in HEK cells. The researchers therefore concluded that they had observed a special BK channel or at least a BK-like channel in LNCaP cells, which they designated BK_L_. However, it remained unknown how the channel's voltage dependence was unprecedentedly shifted to such a great extent, and this could not be readily explained by any previously known modulatory mechanisms, such as alternative splicing, phosphorylation, or β subunits.

Five years later, it was demonstrated that the LNCaP cells did express BKα at the protein level, as detected by the BKα antibody, but the BKα existed in a normal splicing form, according to reverse transcriptase PCR and sequencing of mRNA (Yan and Aldrich, 2010). The authors then took a proteomic approach to immunopurify the channel complex and used mass spectrometry to identify potential novel interacting partners that may drastically modify the BK channel's gating property (Yan and Aldrich, 2010). A 35-kDa leucine-rich repeat-containing protein, LRRC26, was specifically identified in the BKα pull-down components. Knockdown of this protein in LNCaP cells resulted in a complete loss of the BK channel's property of being activated at low voltage in the absence of calcium. Meanwhile, overexpression of LRRC26 in another prostate cancer cell line, PC3, which lacks endogenous LRRC26 expression, converted the endogenous typical BKα channels to the low-voltage–activated LNCaP-type BK channels. It was additionally shown in a heterologous expression system (HEK-293 cells) that LRRC26 was specifically associated with BKα as detected by reciprocal co-immunoprecipitation and shifted the conductance-voltage (G-V) relationship of BK channels to the hyperpolarization direction by ~140 mV, as was seen in the LNCaP cells. This suggests that direct channel complex formation likely occurs without mediation by other proteins.

**Figure 2:**
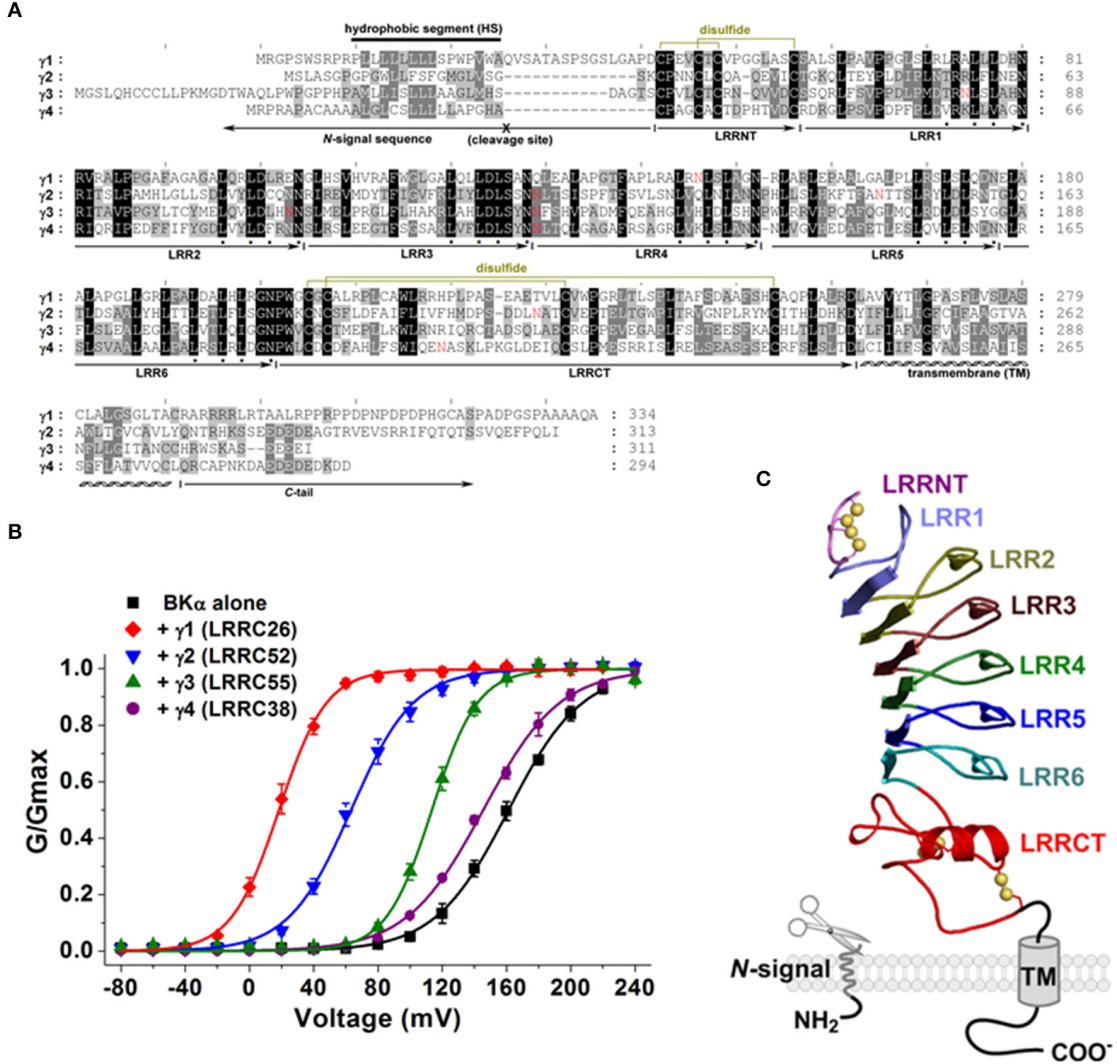
**Structure and function of BK channel γ subunits. (A)** Protein sequence alignment of BK channel γ subunits in humans. The hydrophobic segments and potential cleavage sites in the *N*-terminal signal peptide sequences are indicated. Potential *N*-glycosylation sites of Asn residues are shown in red. Cysteine pairs (total of 4) for potential disulfide formation are also shown. Key residues of the consensus sequence (LxxLxLxxN) in each leucine-rich repeat unit are marked by a filled square at the bottom. **(B)** Modulatory effects of γ subunits on the voltage dependence of BK channel activation in the absence of intracellular calcium, upon heterologous expression of the γ subunit in HEK-293 cells. **(C)** Predicted leucine-rich repeat domain structure and membrane topology of the γ subunit.

LRRC26 is structurally and functionally distinct from the 4β subunits and thus was considered a new type of BK channel auxiliary subunit. Later on, 3 other structurally related leucine-rich repeat-containing proteins, LRRC52, LRRC55, and LRRC38, were reported to be also able to modulate BK channels when co-expressed heterologously with BKα in HEK-293 cells (Yan and Aldrich, 2012) (Figure 2). These proteins have also been shown to produce marked shifts in the voltage dependence of BK channel activation in the hyperpolarization direction, although the shifts are smaller than those produced by LRRC26. LRRC52 causes a shift of approximately 100 mV, LRRC55 causes a shift of 50 mV, and LRRC38 causes a shift of 20 mV in the absence of [Ca^2+^]_i_ (Figure 2B). The ion channel auxiliary subunits, such as the BK or Kv channel β subunits, may exist in multiple paralogous forms with related modulatory functions. These three LRRC proteins, together with LRRC26, have been thus designated as the γ family of the BK channel auxiliary subunits (Yan and Aldrich, 2012). LRRC26 was named γ1 while LRRC52, LRRC55, and LRRC38 were tentatively named γ2, γ3, and γ4, respectively, according to their capabilities to modify the voltage dependence of BK channel activation. Similar to BK β subunits, these proteins also displayed different tissue-specific expression at the mRNA level (Yan and Aldrich, 2012).

## Structural characteristics

The 4γ subunits have similar molecular weights, around 35 kDa. They are type I single-span membrane proteins containing a classic *N*-terminal cleavable signal peptide for extracellular localization of the *N*-terminal LRR domain in the mature proteins. The signal peptide region was found to be absent in the mature protein. Mutations in this region caused the signal peptide to be retained in the expressed protein and led to a loss of modulatory function in the γ1 subunit, suggesting that proper maturation guided by the signal peptide region is critical for the function of γ subunits (Yan and Aldrich, 2012). The mature proteins of the 4γ subunits all contain a single transmembrane domain, an *N*-terminal extracellular LRRD, and a short *C*-terminal tail. The 4γ subunits share an overall sequence similarity of 35–40% (Figure 1A) which are comparable with that among the 4β subunits.

The atomic structure of γ subunits is not yet known. However, a comparison of the amino acid sequences in the LRR domains of γ subunits with those in other LRR-containing proteins provided a good prediction of the structure of γ subunits. The LRR domains of γ subunits all contain 6 LRR units and 2 cysteine-rich regions, a small one called LRRNT, capped on the *N*-terminal side, and a large one called LRRCT, capped on the *C*-terminal side (Figure 2A). As in many other LRR-containing proteins, each LRR unit in the γ subunits consists of 24 residues and has a classic consensus sequence of LxxLxLxxN (where x can be any amino acid). Because no structure has been described for an LRR domain containing both LRRNT and LRRCT elements that are similar to those in the γ subunits, structures of lymphocyte receptor B (Kim et al., 2007a) for the LRRNT and LRR regions and structures of mouse TLR4 (Kim et al., 2007b) for the LRRCT region have been referred to in structural modeling (Yan and Aldrich, 2010, 2012). The resultant structural model for the γ subunit LRR domain can be depicted as in Figure 2C. According to this structural model, the LRR domain is a banana-shaped structure with a curved parallel β-sheet lining the inner circumference and small helices or turns flanking the convex circumference, formed by 6 LRR units stacked together in the middle. Each LRR unit forms a β-strand lining the concave face and a short α-helix connected by loops flanking the outer circumference. The hydrophobic core of the LRR domain is tightly packed by the parallel inward-pointing leucine residues, shielded by the LRRCT and LRRNT caps on the *N*- and *C*-terminal ends. Both LRRNT and LRRCT contain 2 pairs of fully conserved cysteine residues that in total potentially form 4 disulfide linkages in the favorable oxidizing extracellular environment. Consistent with their predicted extracellular location, the LRR domains of the γ subunits all contain single or multiple consensus N-glycosylation sites: Asn-Xaa-Ser/Thr, where Xaa is not a proline. For the γ1 subunit, N147Q mutation and enzymatic removal of the N-linked glycan by PNGase F resulted in disappearance of an upper glycosylated-mass band in SDS-PAGE (Yan and Aldrich, 2012).

The protein sequences in the LRR domains of γ subunits are closely related but become divergent in the transmembrane and intracellular *C*-terminal tail regions (Figure 1A). The single-transmembrane segments of the γ subunits are well predicted from their hydrophobicity and the presence of charged residues on both sides, particularly multiple positively charged residues on the intracellular side following the general “positive-inside rule” for membrane insertion and orientation of membrane proteins. For the *C*-terminal tail regions, in addition to the cluster of positively charged residues adjacent to the transmembrane domain, it is interesting to note that the rest of the amino acid sequence is polyproline (11 proline residues out of 36 residues) for γ1 and polyacidic for γ2, γ3, and γ4.

## Modulatory mechanisms

**Figure 3:**
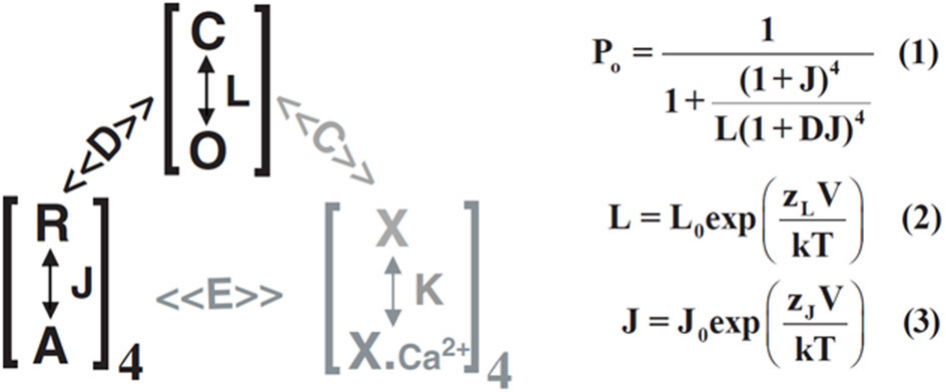
**Schematic and equations of the HA allosteric gating model for BK channel activation by voltage**. The two processes of voltage-sensor activation (equilibrium constant J) and channel opening (equilibrium constant L) are linked by the allosteric coupling factors D. The calcium binding (equilibrium constant K) and the related allosteric coupling factors C, E are shown in gray. P_o_ in the equation (1) means channel open probability.

To date, the structures of the whole γ subunit, as well as the whole BKα channel, have not yet been described. The detailed mechanisms underlying how the γ subunits bind to the BKα tetramer and regulate channel function remain largely unexplored and unknown. The gating shift produced by γ1 subunit is equivalent to the effect of ~10 μM [Ca^2+^]_i_ on BK channels formed by BKα alone. However, Ca^2+^ and Mg^2+^ sensitivities were shown to be largely unaffected by the γ1 subunit (Yan and Aldrich, 2010). Therefore, the mechanistic actions of the γ1 subunit were investigated and analyzed within the framework of the well-established BK channel allosteric model of voltage-dependent gating in the absence of Ca^2+^. According to this HA model (Horrigan and Aldrich, 2002), the activation or open probability (P_o_) of BK channels by voltage can be simply calculated or described by 5 gating parameters: *L*_0_ and *J*_0_ together with *Z*_L_ and *Z*_J_ were referred to as equilibrium constants and associated gating charges for the channel pore's closed ↔ open and voltage sensors' resting ↔ activated transitions and *D* was considered the allosteric coupling factors between the pore and the voltage sensors (Horrigan and Aldrich, 2002) (Figure 3). Using simulations, and by measuring the kinetics and open probabilities of the channels at very negative voltages to achieve good estimates of the *Z*_L_ and *L*_0_ parameters, it was found that the γ1 subunit's modulatory effect can be best explained by a ~20-fold increase in the allosteric coupling *D* factor, whereas the pore's gating parameters *L*_0_ and *Z*_L_ are largely unaffected by the γ1 subunit (Yan and Aldrich, 2010). According to this study, the γ1 subunit may mainly affect the coupling between voltage sensors and the pore. However, the possibility cannot be ruled out that the γ1 subunit also slightly affects *Z*_J_, *J*_0_ or *L*_0_, owing to limitations in the accuracy of experimental data obtained at very negative voltages and the assumption used in simulations that only one gating parameter is affected. The γ1 subunit may also act on some other aspect of BK channel gating, such as the two distinct calcium binding events on RCK1 and RCK2 domains (Xia et al., 2002) and the interactions among the two calcium binding sites and the voltage sensor (Qian et al., 2006; Sweet and Cox, 2008; Savalli et al., 2012), which were not addressed by HA model (Horrigan and Aldrich, 2002) and also excluded for consideration in the modeling analyses of the γ1 modulatory effect (Yan and Aldrich, 2010). Regardless of these limitations, γ subunits may serve as good tools to study BK gating mechanisms because little is currently known about the molecular basis underlying allosteric coupling between voltage sensors and the pore in this voltage- and ligand-gated channel.

The action of the γ1 subunit is remarkable in its modulatory magnitude and mechanistic simplicity. A recent study indicated that the regulatory mechanism of the γ1 subunit may be fundamentally different from that of the β subunit (Gonzalez-Perez et al., 2014). In contrast to β subunits, which have the ability to regulate the voltage dependence of BK channel activation in the titration-dependent mode, the γ1 subunit exhibited an “all-or-none” regulatory pattern (Gonzalez-Perez et al., 2014). In a classic model, it is understood that auxiliary subunits bind to the BKα subunit one by one with 4-fold symmetry so that the regulatory effect is incremental (Wang et al., 2002). However, the γ1 subunit caused the voltage dependence of channel activation to be either shifted to a full extent or unchanged independent of the molar ratio of the injected BKα:γ1 RNA to *Xenopus oocytes*, although the ratio of these two populations of channels varied (Gonzalez-Perez et al., 2014). It is unknown whether one γ1 subunit per channel complex is sufficient to fully modulate BK channels. Alternatively, the γ1 subunit may preferably exist in a tetrameric form when forming a complex with BKα. Additional studies will be required to determine the detailed mechanisms, particularly the stoichiometry and the interaction sites between the BKα and γ subunits in the tetrameric channel complex.

Not surprisingly, the γ1 subunit can inhibit the effect of some BK activators (Almassy and Begenisich, 2012). The endogenous γ1 subunit was present in native salivary gland parotid acinar cells; it blocked the activating effect of mallotoxin (MTX) but not NS-1619 (Almassy and Begenisich, 2012). Similar blocking effect of the γ1 subunit on MTX action was also observed in HEK-293 cells when the γ1 subunit was heterologously co-expressed with BKα. It was proposed that MTX may displace the γ1 subunit instead of lacking accessibility to the binding site (Almassy and Begenisich, 2012). Further biophysical studies and biochemical binding assays will be needed to clarify the detailed mechanisms. MTX and γ1 are likely exclusively and sterically related in their binding to the BK channels. NS1619 was recently shown to bind to the S6/RCK linker region (Gessner et al., 2012), but little is currently known about the MTX binding site. Identification of the MTX binding site may complement our understanding of the actions of γ subunits on BK channels.

## Physiologic relevance

The tissue-specific distribution patterns of the 4γ subunits at the mRNA level had been investigated using TaqMan quantitative PCR in various human tissues (Yan and Aldrich, 2012). The γ1 subunit was highly expressed in the salivary glands, prostate, and trachea, whereas γ2 (LRRC52) was found predominantly in the testes and γ3 (LRRC55) was found primarily in the nervous system. The γ4 (LRRC38) subunit was mainly observed in skeletal muscle, adrenal glands, and the thymus. These results suggest that like β subunits, γ subunits have different tissue-specific distributions to fit the diverse functional requirements of various tissues and cell types (Yan and Aldrich, 2012). The γ1 subunit's endogenous functional regulation of BK channels has been confirmed in prostate and salivary gland cells (Yan and Aldrich, 2010; Almassy and Begenisich, 2012). The physiological roles of the γ1 subunit in prostate and salivary glands remain to be determined. Conceivably, constitutive activation of BK channels might be required for K^+^ flow-mediated fluid secretion in these non-excitable tissues. A very recent study implies that the γ1 subunit in airway epithelial cells may participate in the BK channel-mediated airway hydration for effective mucociliary clearance (Manzanares et al., 2014). The K^+^ flow through the apically expressed BK channels in airway epithelial cells provides an electrochemical driving gradient for Cl^−^ secretion and thus plays a role in airway hydration. It was found that both the mRNA level of the γ1 subunit and the sensitivity of BK channels to mallotoxin were decreased after IFN-γ treatment, implying that the γ1 subunit might be involved in the IFN-γ-mediated reduction in BK channel activity and the resulting mucociliary dysfunction (Manzanares et al., 2014). The γ1 subunit under a different name (CAPC) was reported to be able to suppress tumor growth and metastasis, which may likely involve ion channel–independent function (Liu et al., 2012). It will be necessary to determine how the association of the γ1 subunit with BK channels may affect tumor growth because of the enhanced K^+^ channel activity that generally promotes cancer cell proliferation (Pardo and Stuhmer, 2014).

Because of the drastic activating effect caused by the γ1 subunit, expression of this protein even at low levels might exert significant effect on BK channel currents. For example, a low mRNA expression level of the γ1 subunit has been detected in aorta cells (Yan and Aldrich, 2012). A very recent study reported that knockdown of γ1 subunit expression in rat cerebral artery myocytes led to a reduction in the apparent voltage /Ca^2+^ sensitivity and current frequency and amplitude of BK channels, as well as a decrease in the extents of BK channel-specific inhibitor-induced vasoconstriction and activator-induced vasodilation (Evanson et al., 2014). This study suggests that the γ1 subunit may play broad physiological roles that are not limited to non-excitable cells. In excitable cells, the voltage and Ca^2+^ sensitivities of the BK channels are more finely tuned to be properly responsive to different levels of voltage and Ca^2+^ in different cell types, and therefore low expression of this potent BK channel modulator might exert a significant physiological effect. It is worth noting that the γ1 subunit is also expressed in fetal brain tissue (Yan and Aldrich, 2012), and the γ1 subunit might participate in maintaining proper neuronal excitability in the fetal nervous system during early development.

## Perspectives

Our understanding of BK channel γ subunits is still in its infant stage. In particular, very little is known about the physiologic functions and the structural basis underlying the regulatory mechanisms of γ subunits. The discovery of γ subunits adds a new dimension to BK channel regulation and provides a molecular basis for a better understanding of the physiological functions of BK channels in different tissues or cell types. The few published studies examining the modulatory mechanisms and physiological functions of BK channel γ subunits have mainly focused on γ1, the most potent γ subunit. The regulation of BK channels by γ2-4 subunits has so far been demonstrated only in the heterologous expression system. It will be important to determine whether γ2-4 subunits also play any functional or physiological role in BK channel modulation *in vivo*.

The γ2 subunit is also potent in shifting the voltage dependence of BK channel activation. The presence of the γ2 subunit specifically in the testes among the examined mouse and human tissues suggests that it plays a special role in spermatogenesis or male fertility (Yang et al., 2011; Yan and Aldrich, 2012). A detailed study suggested that the mouse γ2 subunit functions as an accessory subunit of the sperm-specific mouse Slo3 channels (Yang et al., 2011). Co-expression of Slo3 and γ2 in *Xenopus* oocytes generated pH and voltage-dependent currents that are more similar to native KSper than those of the Slo3 channel alone. Moreover, Slo3 deletion in mouse testes and sperm either significantly decreased or eliminated the expression of γ2. In rats, BK channel-like currents and immunostaining of BKα was found to be high in premeiotic germ cells, spermatozoa and primary spermatocytes, but very low in postmeiotic germ cells (Gong et al., 2002). It will be intriguing to determine whether the γ2 subunit also modulates BK channels and whether Slo3 and BK channels can form functional heterotetrameric channels in any stage of germ cells. BK channels and Slo3 belong to the Slo channel family, which also includes 2 more distantly related Na^+^-activated channels, Slo2.1 (slick) and Slo2.2 (slack). There is currently no report on the effect of γ subunits on the Slo2.1 or Slo2.2 channels. It remains an open question whether the γ subunits may broadly function as auxiliary proteins of the Slo channel family.

Effective BK channel openers have been sought or explored to treat a variety of diseases such as stroke, epilepsy, psychoses, bladder overactivity, erectile dysfunction, asthma, arterial hypertension, ischemic heart disease, and gastric hypermotility (Nardi and Olesen, 2008). Although the widely used BK channel opener NS1619 can give ~-40-mV shift in V_1/2_ at a high concentration (30 μM) (Gessner et al., 2012), its specificity was recently questioned because of its direct inhibiting effect on the sarco/endoplasmic reticulum Ca^2+^-ATPase (SERCA) (Wrzosek, 2014). The γ1 subunit, which is so far the most potent BK channel activator, and the other γ subunits of different potencies provide molecular tools to manipulate BK channel activity *in vivo* through either transgenic or viral delivery of gene expression. This may offer the opportunity to evaluate the therapeutic potential of BK channel activators (openers) of different channel-activating potencies in the treatment of various diseases. Currently, no BK channel–targeted drug has been approved for clinical use in spite of extensive academic and pharmaceutical efforts over the past two decades. Deciphering the biochemical mechanisms underlying BK channel activation by γ subunits will be very useful for the development of new BK channel–targeted drugs.

### Conflict of interest statement

The authors declare that the research was conducted in the absence of any commercial or financial relationships that could be construed as a potential conflict of interest.
